# A systematic overexpression approach reveals native targets to increase squalene production in *Synechocystis* sp. PCC 6803

**DOI:** 10.3389/fpls.2023.1024981

**Published:** 2023-05-30

**Authors:** Anna T. Germann, Andreas Nakielski, Maximilian Dietsch, Tim Petzel, Daniel Moser, Sebastian Triesch, Philipp Westhoff, Ilka M. Axmann

**Affiliations:** ^1^ Institute for Synthetic Microbiology, Department of Biology, Heinrich Heine University Düsseldorf, Düsseldorf, Germany; ^2^ Institute for Plant Sciences and Cluster of Excellence on Plant Sciences (CEPLAS), University of Cologne, Cologne, Germany; ^3^ Institute of Plant Biochemistry, Cluster of Excellence on Plant Science (CEPLAS), Heinrich Heine University, Düsseldorf, Germany; ^4^ Plant Metabolism and Metabolomics Laboratory, Cluster of Excellence on Plant Sciences (CEPLAS), Heinrich Heine University Düsseldorf, Düsseldorf, Germany

**Keywords:** Synechocystis, squalene, MEP pathway, FBA, metabolic engineering, metabolic modeling

## Abstract

Cyanobacteria are a promising platform for the production of the triterpene squalene (C30), a precursor for all plant and animal sterols, and a highly attractive intermediate towards triterpenoids, a large group of secondary plant metabolites. *Synechocystis* sp. PCC 6803 natively produces squalene from CO_2_ through the MEP pathway. Based on the predictions of a constraint-based metabolic model, we took a systematic overexpression approach to quantify native *Synechocystis* gene’s impact on squalene production in a squalene-hopene cyclase gene knock-out strain (Δ*shc*). Our *in silico* analysis revealed an increased flux through the Calvin-Benson-Bassham cycle in the Δ*shc* mutant compared to the wildtype, including the pentose phosphate pathway, as well as lower glycolysis, while the tricarboxylic acid cycle predicted to be downregulated. Further, all enzymes of the MEP pathway and terpenoid synthesis, as well as enzymes from the central carbon metabolism, Gap2, Tpi and PyrK, were predicted to positively contribute to squalene production upon their overexpression. Each identified target gene was integrated into the genome of *Synechocystis* Δ*shc* under the control of the rhamnose-inducible promoter P_rha_. Squalene production was increased in an inducer concentration dependent manner through the overexpression of most predicted genes, which are genes of the MEP pathway, *ispH*, *ispE*, and *idi*, leading to the greatest improvements. Moreover, we were able to overexpress the native squalene synthase gene (*sqs)* in *Synechocystis* Δ*shc*, which reached the highest production titer of 13.72 mg l^-1^ reported for squalene in *Synechocystis* sp. PCC 6803 so far, thereby providing a promising and sustainable platform for triterpene production.

## Introduction

Cyanobacteria are the only known prokaryotes capable of oxygenic photosynthesis (Mulkidjanian et al., 2006; Lau et al., 2015). The gram-negative bacteria exhibit a large ecological variety as well as a broad morphological diversity (Bennett and Bogorad, 1973; Rippka et al., 1979; Schirrmeister et al., 2013). Their physiological diversity makes them promising biological chassis for the synthesis of a variety of natural products, including bioactive metabolites like cytotoxins and potential pharmaceutical lead compounds, food supplements, animal feed, pigments, as well as biofuels (Pulz and Gross, 2004; Hays and Ducat, 2015; Jain et al., 2017; Hudson et al., 2021; Barone et al., 2023). Their ability to convert sunlight and atmospheric CO_2_ directly into valuable organic compounds could make the chemical and pharmaceutical industry more sustainable and therefore mitigate climate change if high production yields are achieved (Choi et al., 2020; Posten and Schaub, 2009; Oliver and Atsumi, 2015; Oliver et al., 2016).

Metabolic engineering tools for the production of desired compounds are particularly well established for laboratory model strains like *Synechocystis* sp. PCC 6803 (hereafter *Synechocystis*) (Knoop and Steuer, 2015; Ramey et al., 2015). The unicellular organism was the first entirely sequenced cyanobacterium (Kaneko et al., 1996) and is one of the best characterized model organisms regarding cyanobacterial biosynthesis (Pils and Schmetterer, 2001; Leplat et al., 2013). *Synechocystis* is easy and inexpensive to cultivate, and is genetically modifiable with high success rates and predictability (Rippka et al., 1979; Berla et al., 2013).

One promising class of compounds to be produced in cyanobacteria are terpenoids, a large heterogeneous group of naturally occurring organic carbon compounds with over 80,000 known structures (Karunanithi and Zerbe, 2019), having applications in nutrition, medicine and chemistry but also as potential biofuels. Triterpenoids are a group of secondary metabolites, which are composed of 6 isoprene units (Moss et al., 1995), existing in a huge variety of structures with nearly 200 distinct triterpene skeletons, all deriving from the precursor squalene (Xu et al., 2004; Connolly and Hill, 2010). Squalene is typically extracted from shark liver oil, but this method poses serious ecological risks and is not sufficient to sustainably meet increasing demands (Gohil et al., 2019; O’Hagan et al., 2021; Mendes et al., 2022). The diverse applications of squalene include its use as an ingredient in cosmetic products (Gohil et al., 2019), as an antioxidant (Kohno et al., 1995) and an emulsion adjuvant in vaccines (O’Hagan et al., 2021). It has recently been used in several COVID-19 vaccines (Liang et al., 2020; Ho et al., 2021), introducing a surge in demand for this terpenoid. Other reported properties of squalene include tumor-suppressing (Smith et al., 1998; Yang et al., 2014), immunity improving (Ronco and De Stéfani, 2013), cholesterol-lowering (He et al., 2003), as well as antibacterial and antifungal effects (Katabami et al., 2015). Squalene has attracted attention as a feasible source of biofuels (Hellier et al., 2013) as well, if it could be produced sustainably and in large quantities.

In most plants, algae and prokaryotes, terpenoids can be synthesized via the methyl-erythritol-4-phosphate (MEP) pathway, also called the non-mevalonate pathway (Okada and Hase, 2005; Sawai and Saito, 2011). While the mevalonate pathway is present in most eukaryotic cells, the MEP pathway was acquired through endosymbiotic or horizontal gene transfer in plastid-bearing organisms. As the progenitors of plastids, cyanobacteria can serve as both a model organism for chloroplastic terpenoid synthesis through the MEP pathway and are promising production hosts for plant terpenoids (Hemmerlin et al., 2006; Loeschcke et al., 2017). The MEP pathway produces isopentenyl diphosphate (IPP) and dimethylallyl diphosphate (DMAPP), which are the universal precursors for terpenoid synthesis. In *Synechocystis*, a single gene, *crtE*, is responsible for the elongation of terpene precursors towards geranyl pyrophosphate (GPP), farnesyl pyrophosphate (FPP) and geranylgeranyl pyrophosphate (GGPP) in consecutive condensation reactions. The enzyme squalene synthase (Sqs) catalyzes the condensation of two molecules of FPP to presqualene diphosphate (PSPP), which is then converted into squalene via reduction by NADPH. In *Synechocystis*, squalene is then cyclized by squalene hopene cyclase (Shc) to hopene (Englund et al., 2014). Englund and colleagues (Englund et al., 2014) used a modified strain of *Synechocystis* to produce squalene by inactivating the *shc* gene. By preventing the generation of hopene, squalene is accumulated in this mutant. We previously constructed a markerless deletion of *shc* in *Synechocystis* to minimize the number of antibiotic resistances carried by each strain and enable further engineering (Dietsch et al., 2021). The availability of inducible promoter systems, such as the rhamnose, anhydrotetracycline or copper inducible promoters in *Synechocystis* allows fine-tuning of gene expression levels, making improvement of metabolic pathways possible by identifying optimal expression levels for each involved gene (Behle et al., 2020).

A straightforward approach to achieve higher yields of desired products is the overexpression of certain genes to increase the flux towards these metabolites, with many strategies already reported for increasing heterologous terpenoid production (Klaus et al., 2022). Despite some strategies proving successful, the regulation and bottlenecks of the MEP pathway are still not entirely understood (Klaus et al., 2022). Most initial pathway modification approaches result in relatively low product yield, and optimization is often dependent on heuristic techniques (Choi et al., 2017; Steuer et al., 2012). While the identification of genes to be modified is an essential step in metabolic engineering for strain improvement toward the enhanced production of desired bioproducts, it is still difficult to decide which genes to insert or modify, due to the vast number of possibilities of potential targets and the consideration of complex regulation of metabolic networks. In order to rationally identify and overcome bottlenecks for the improvement of strain designs, the use of *in silico* models has gained increasing significance over the past years (King et al., 2015; Broddrick et al., 2016; Lin et al., 2017; Hendry et al., 2020). Genome-scale metabolic modeling requires only stoichiometric information and no kinetic parameters, which are usually not available even for small reaction networks. In contrast, stoichiometric information is readily and reliably available for a large number of annotated genes (Raman and Chandra, 2009). Additionally, the estimation of stoichiometric yield is computationally not expensive and feasible even for large models involving hundreds of reactions (Knoop and Steuer, 2015). Constraint-based reconstruction and analysis (COBRA) utilizes these models to calculate flux distributions under certain environmental or internal conditions (Schellenberger et al., 2011). COBRA methods are mainly used for the prediction of maximum theoretical yield for native and non-native pathways (Shen and Liao, 2013; King et al., 2015), the effect of gene deletions on biomass or other target compounds (e.g. OptKnock) (Burgard et al., 2003) as well as the identification of bottlenecks and potential targets for up- and downregulation in order to increase product yield (e.g. flux scanning based on enforced objective flux (FSEOF) or minimization of metabolic adjustment (MOMA)) (Segrè et al., 2002; Choi et al., 2010). Previous studies (Choi et al., 2010; Englund et al., 2018; Park et al., 2018), aiming to increase terpenoid production, have shown that constraint-based flux balance analysis (FBA) can be a helpful tool to not only understand and analyze metabolic pathways, but to identify bottlenecks and narrow down the options of potential amplification or knock-down targets for increased product yield, without having a negative influence on the growth rate. Englund et al. successfully employed genome-scale metabolic flux analysis to identify amplification targets that increased isoprene production in *Synechocystis* (Englund et al., 2018).

In our work, we used an algorithm called FSEOF to screen for potential overexpression targets increasing the terpenoid concentration in *Synechocystis* (Choi et al., 2010). *In silico* analysis predicted an increased flux through reactions of the MEP pathway, terpenoid synthesis, the light-dependent reactions of photosynthesis as well as the central carbon metabolism. Additionally, metabolic modeling proposed elevated requirements for the efficient supply, balance and regeneration of cofactors. Twelve of the identified amplification targets were tested *in vivo* to assess the biological relevance of the model, all of which positively impacted squalene production. The overexpression of *sqs* led to the strongest increase, with a yield of 13.72 mg l^-1^, the highest production titer reported for squalene in *Synechocystis* to date.

## Materials and methods

### Plasmid and strain construction

A detailed list of all relevant genetic modules and information regarding their origin, is provided in the Supporting Information (**Table S1** (SI)).

To investigate the computationally identified genes’ effect on squalene production, the pEERM4 plasmid was used to integrate each gene into the neutral site 2 (NS2) under control of the rhamnose promoter P_rha_ (Englund et al., 2015; Behle et al., 2020). The plasmid pEERM4 Cm was a gift from Pia Lindberg (Addgene plasmid # 64026; http://n2t.net/addgene:64026; RRID : Addgene_64026) (Englund et al., 2015). This plasmid was used to clone each gene of interest under the control of P_rha_. It contains 500 bp DNA homologous to the upstream and downstream region of NS2, between which a chloramphenicol resistance and the gene of interest are located, flanked by the rhamnose promoter and the T7 terminator. Each gene of interest was cloned into the plasmid using the restriction enzymes *Nhe*I and *Pst*I, with the *Nhe*I cutting site located after the start codon. The genes of interest were amplified from the *Synechocystis* genome, using Q5-Polymerase (NEB # M0491) according to manufacturer’s instructions with oligonucleotides shown in **Table S2** (SI). In two cases, an NheI restriction site was removed from the native gene sequence without changing the amino acid sequence (*gap2, sqs*). The *sqs* gene is annotated as starting with GTG as a start codon in the published Kazusa genome and this codon was changed to ATG for the purposes of this study.

To enable induction of the P_rha_ promoter, the rhamnose activator rhaS was constitutively expressed by the J23119 promoter from the replicative plasmid pSHDY (AddGene Plasmid #137661, (Behle et al., 2020)), which was transferred to *Synechcoystis* via triparental mating (Behle et al., 2020). This plasmid was constructed using the restriction sites of the BioBrick and NeoBrick standards and carries a spectinomycin resistance.

*Synechocystis* was transformed with the pEERM4 plasmids (**Table S1** (SI)) using a protocol based on its natural competence (dx.doi.org/10.17504/protocols.io.mdrc256). Successful integration of the plasmid into the genome through heterologous recombination into the neutral site 2 (NS2) (Satoh et al., 2001) was verified by colony PCR (**Figure S1** (SI)). The plasmid pSHDY carrying the rhamnose activator *rhaS* was then transferred to *Synechocystis* using triparental mating (dx.doi.org/10.17504/protocols.io.psndnde).

### Culture conditions

The *Synechocystis* strains were inoculated in 30 ml BG11 liquid cultures containing 20 µg/ml spectinomycin and for overexpression strains 10 µg/ml chloramphenicol in 100 ml Erlenmeyer flasks from agar plates. The cultures were diluted twice to an OD_750_ of 0.2 to equalize their cell densities and growth phases. Two days before the start of the experiment, cultures were again diluted to an OD_750_ of 0.2 after which they were transferred to 6-well plates with 5 ml per well. L-Rhamnose was then added to the cultures and they were grown for 72 h at 30°C with 150 rpm shaking, 0.5% CO_2_ and 80 µE m^-2^ s^-1^ of continuous light. After 72 h, cell samples were taken and stored for further processing.

For measurements of squalene production over time, 30 ml of BG11 were inoculated from a pre-culture to OD_750 = _0.4, supplemented with 5 mM of rhamnose and incubated over 14 days. Samples were taken daily for the first four days, every second day for the following six days and after 14 days. The lost culture volume from sampling was replaced with fresh BG11 containing appropriate antibiotics and 5 mM of rhamnose.

### Biomass measurements (DCW, OD, spectra)

Cell dry weight measurements were carried out by transferring the cell pellet of 2 ml of cyanobacterial culture to a pre-weighed PCR tube, which was incubated at 60°C for 20 h. The tube was weighed and the difference noted as the cell dry weight, with measurements carried out in triplicates.

Absorption spectra and OD measurements were carried out in 1 ml polystyrene cuvettes in a SPECORD 200 Plus Spectrophotometer (Analytik Jena) with BG11 as a blank and as a reference sample. Samples were diluted with BG11 to be within an absorption range of 0.1 to 1.0 to ensure accurate measurements. Cell densities for *Synechocystis* were measured at 750 nm.

### Pigment quantification

Each culture (300 µL) was sampled after 72 hours at the end of the growth experiment. The sample was centrifuged at 14,000 g for 5 minutes and 4°C. The supernatant was discarded and the pellet was resuspended in 100 μl water. The samples were frozen at -80°C until further processing. 900 μl of 100% methanol were added to the sample and the sample was mixed by vortexing. After incubation in the dark under gentle agitation for 1 h at 4°C the sample was centrifuged at 14,000 g for 5 minutes. The supernatant was transferred into a cuvette and an absorbance spectrum was measured from 400 nm to 750 nm. The absorbance spectra were divided by the OD_750_ or CDW and the amount of chlorophyll *a* in the sample was quantified by the absorbance maximum of chlorophyll *a* at 665 nm (A_665nm_) using following equation (Lichtenthaler and Buschmann 2001):


$$\text{Chlorophyll content}\left[\text{μg}/\text{ml}\right]=12.66\text{ μg}/\text{ml}*{\text{A}}_{665\;\text{nm}}$$

The amount of carotenoids in the sample was quantified by the absorbance maximum of the sum of carotenoids at 470 nm (A_470nm_) and a correction term considering absorbance of chlorophyll *a* at 470 nm (c(Chl *a*): concentration of chlorophyll *a* in the sample) using following Equation (Lichtenthaler and Buschmann 2001):


$$\text{Carotenoid content}\left[\text{mg}/\text{ml}\right]=\left(1000\text{ μg}/\text{ml}*{\text{A}}_{470\;\text{nm}}-1.91*\text{c}\left(\text{Chl}\right)\right)/225$$

### GC-MS measurements for the quantification of squalene

Each culture (1.5 ml) was sampled after 72 hours at the end of the growth experiment. The sample was centrifuged at 14,000 g, for five minutes and 4°C. The supernatant was discarded and the pellet was frozen at -80°C until further processing. The pellet was extracted with 500 µL acetone, containing 25 µM β-sitosterol as internal standard, under agitation at 1000 rpm and 50°C for 10 min. 500 µL of 1 M NaCl was added and mixed by vortexing. After adding 250 µL hexane, the sample was vigorously mixed for 1 min and centrifuged for phase separation (1 min at 1,780 g and 4°C). The upper hexane phase was transferred into GC-MS vials and stored at -20°C until the analysis.

GC-MS analysis was carried out using a Gerstel automatic liner exchange system with multipurpose sample MPS2 dual rail and two derivatization stations, used in conjunction with a Gerstel CIS cold injection system (Gerstel, Muehlheim, Germany). For every 10-12 samples, a fresh multibaffled liner was inserted. Chromatography was performed using the Agilent 7890B GC. Metabolites were separated on an Agilent HP-5MS column (30ml x 0.25mm), the oven temperature was ramped with 12.5 °C/min from 70 °C (initial temp for 2 min) to 320 °C (final temp hold 5 min). Metabolites were ionized and fragmented in an EI source (70V, 200 °C source temp) and detected using 7200 accurate mass Q-TOF GC-MS from Agilent Technologies. Data analysis was performed using Agilent MassHunter Quantitative Analysis B.09.00. Peaks were identified using already available EI-MS fragmentation data. Peaks were identified using characteristic fragment ions (Bhatia et al., 2013) and retention times of standards (Squalene: mass/charge (m/z) = 81.07, retention time (RT) = 9.5 min; β-sitosterol: m/z = 107.09, RT = 13.6 min). Squalene concentrations in the measured samples were calculated using a calibration curve with a squalene standard (**Figure S2** (SI)).

### Quantitative real-time PCR (qRT-PCR)

Cultures were sampled (0.5 ml) after 72 hours at the end of the growth experiment. The pellet was processed for RNA extraction using the PGTX method (dx.doi.org/10.17504/protocols.io.jm3ck8n, Pinto et al., 2009). The remaining DNA in the extracted RNA was removed by DNase digestion using the TURBO DNA-free™ (ThermoFischer) kit according to the manufacturer’s instructions. Extracted RNAs (250 ng) were used in a reverse transcriptase reaction using the RevertAid First Strand cDNA Synthesis Kit (ThermoFischer) according to the manufacturer’s instructions. The resulting cDNA was diluted 1:20. For performing qPCR, the DyNAmo ColorFlash SYBR Green qPCR Kit was used according to the manufacturer’s instructions. Primers for *sqs*, *dxs* and the housekeeping gene *rpoA* are shown in **Table S2** (SI). Primer efficiencies were tested before performing qRT-PCR and were deemed sufficient to yield quantitative information (**Figure S3**; **Table S3** (SI)). Changes in gene expression as fold changes compared to the control were determined using the 2^−ΔΔCT^ method, using *rpoA* as a housekeeping gene and the Δ*shc* strain subjected to the same rhamnose concentration as a control.

### Metabolic modeling for the identification of amplification targets

All simulations are based on a genome-scale stoichiometric network model of *Synechocystis* published by Knoop and colleagues (Knoop and Steuer, 2015). A modified, extended version was used, kindly provided by Ralf Steuer. All flux distributions have been calculated with constraint-based flux analysis using COBRApy (v.0.25.0) (Ebrahim et al., 2013). To simulate phototrophic growth, different constraints were applied to the model of *Synechocystis* (see **Table S5** (SI)).

FSEOF (Choi et al., 2010) was used to find amplification targets by simulating the transition from a wildtype to a production phenotype. All isoreactions were excluded for the transition experiments (Knoop and Steuer, 2015). The initial fluxes of all reactions were calculated by using the objective function to maximize the growth rate. Then, the theoretical maximum squalene production rate was calculated by setting the objective function as maximizing squalene flux. Subsequently, under constant light flux, the product formation flux rate was stepwise increased from 0% to 67% of the maximum achievable rate, while the growth rate was maximized. Only targets for which the overall mean flux rate from maximum biomass synthesis to maximum product synthesis increases were chosen. Additionally, only reactions that did not change flux direction during transition were considered. To confirm the results, flux variability analysis was performed for the selected targets, by stepwise increasing squalene flux from 0% to 67% of the maximum rate and subsequently maximizing biomass synthesis. For each simulation step, the variability of all selected targets was determined. To visualize the flux distributions a simplified network was implemented with d3flux (v.0.2.7) (St. John, 2016), a d3.js based visualization tool for COBRApy models.

## Results

### Metabolic modeling predicts overexpression targets in MEP pathway and central carbon metabolism

In this study our goal was to enhance squalene production by identifying potential amplification targets and systematically overexpressing selected genes of interest. Due to the extensive number of possible genes to modify, we chose to first screen for suitable targets *in silico*. We used a genome-scale metabolic network of *Synechocystis* to find the ideal flux distribution for the optimal production of squalene while maintaining at least 1/3 of the growth rate. Reactions that increase in flux, when more squalene is produced, were chosen as potential amplification targets.

**Figure 1:**
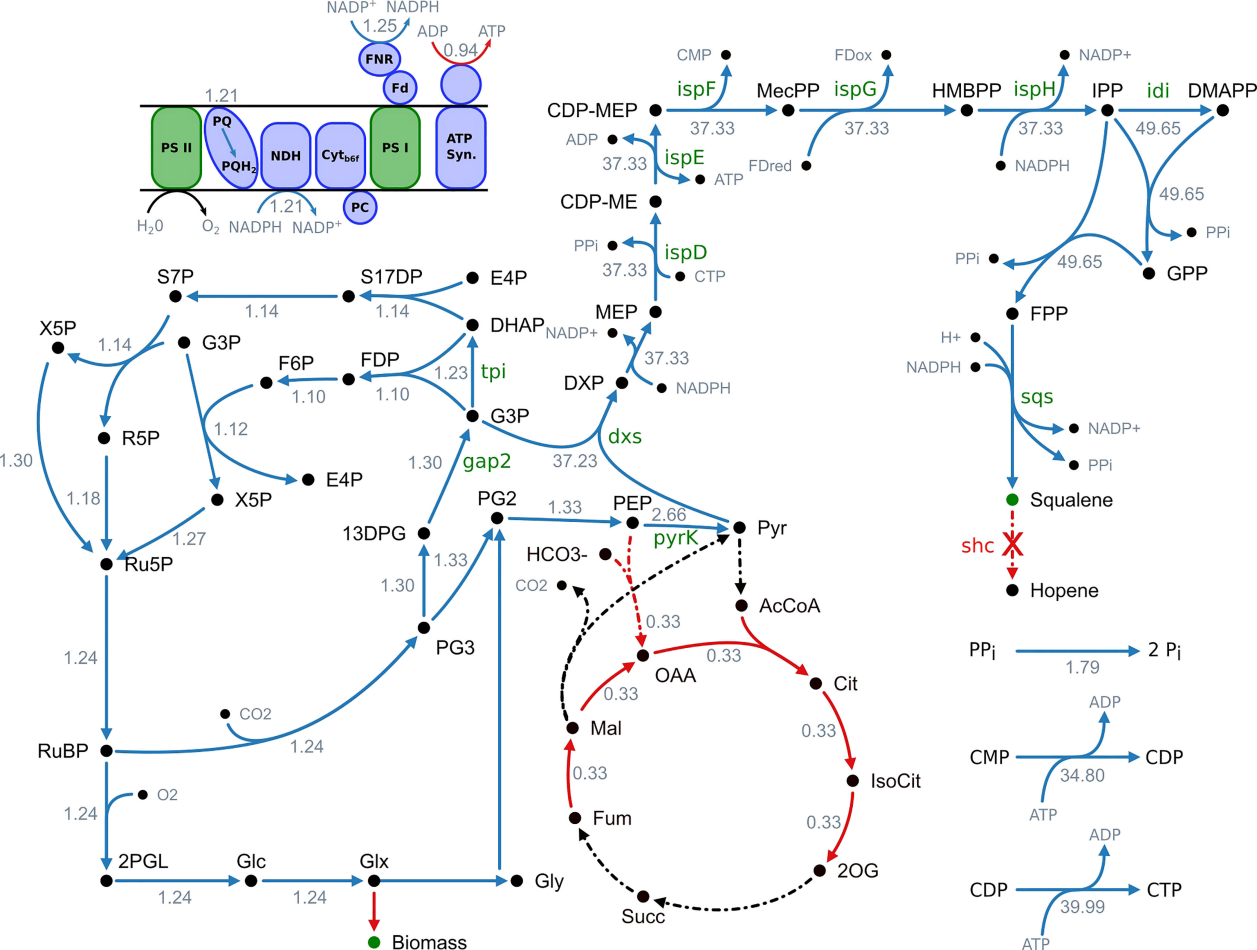
Overview of fluxes predicted to change upon increased squalene production. Blue arrows indicate an increased flux and red arrows a decreased flux, respectively. Black arrows indicate no change. Reactions with no flux have a dotted line. The numbers indicate the maximum fold change of the corresponding flux. It is stated that this is not a minimal network but a part of the genome-scale model and not all active reactions are shown. 13DPG, 1;3-bisphosphoglycerate; 2OG, 2-oxoglutarate; 2PGL, 2-phosphoglycolate; AcCoA, acetyl-CoA; ATP synth., ATP synthase; CDP-ME, 4-(cytidine 5′-diphospho)-2-C-methyl-D-erythritol; CDP-MEP, 2-phospho-4-(cytidine 5′-diphospho)-2-C-methyl-D-erythritol; Cit, citrate; Cyt_b6f_, cytochrome b_6_f complex; DHAP, dihydroxyacetone phosphate; DMAPP, dimethylallyl diphosphate; DXP, 1-deoxy-D-xylulose 5-phosphate; E4P, erythrose 4-phosphate; F6P, fructose 6-phosphate; Fd_ox_, ferredoxin (oxidized); Fd_red_, ferredoxin (reduced); FDP, fructose 1;6-biphosphate; FNR, ferredoxin-NADP^+^ reductase; FPP, farnesyl pyrophosphate; Fum, fumarate; G3P, glyceraldehyde 3-phosphate; Glc, D-glycerate; Glx, glyoxylate; Gly, glycolate; GPP, geranyl pyrophosphate; HMBPP, 1-hydroxy-2-methyl-2-(E)-butenyl 4-diphosphate; IsoCit, isocitrate; IPP, isopentenyl diphosphate; Mal, malonate; MEcPP, 2-C-methyl-D-erythritol 2;4-cyclodiphosphate; MEP, 2-C-methyl-D-erythritol 4-phosphate; NDH, NADPH dehydrogenase; OAA, oxaloacetate; PC, plastocyanin; PEP, phosphoenolpyruvate; PG2, 2-phosphoglycerate; PG3, 3-phosphoglycerate; Pi, orthophosphate; PPi, diphosphate; PQ, plastoquinone; PQH_2_, plastohydroquinone; PSI, photosystem I; PSII, photosystem II; Pyr, pyruvate; R5P, ribose 5-phosphate; Ru5P, ribulose 5-phosphate; RuBP, ribulose 1;5-biphosphate; S17DP, sedoheptulose 1;7-bisphosphate; S7P, sedoheptulose 7-phosphate; Succ, succinate; X5P, xylulose 5-phosphate.

To predict these targets, we applied an algorithm called FSEOF, developed by Choi and colleagues (Choi et al., 2010). The transition from a wildtype phenotype to a production phenotype was simulated by stepwise increasing squalene flux while the growth rate was maximized. This way, all resources beyond the growth rate are directed towards the forced product synthesis. We only considered targets for which the overall mean flux rate increases and that did not change flux direction during transition (Knoop and Steuer, 2015). An overview of reactions meeting these criteria can be found in **Figure 1** and in **Table S6** (SI). Excluding transport, export and spontaneous reactions, 39 potential overexpression targets were identified. Flux variability analysis was performed for these targets to validate the results (**Table S7** (SI)). Upon enforced objective flux different flux patterns could be observed. 22 fluxes showed an increasing pattern without any variability. Among the remaining fluxes two increased within a narrow range and seven increased with broad variability. Three showed a pattern, where the minimal flux increased and the maximum possible flux decreased. One reaction showed a pattern indicating a change in flux direction and four were unbound.

The results of the metabolic modeling suggest an increase of flux through the MEP pathway, from the decarboxylative carboligation of glyceraldehyde 3-phosphate (G3P) and pyruvate to deoxyxylulose 5-phosphate (DXP) by deoxyxylulose 5-phosphate synthase (Dxs), to the formation of isopentenyl diphosphate (IPP) by IspH as well its conversion to DMAPP by isomerization (Idi). The following reactions towards terpenoid synthesis, catalyzed by CrtE and Sqs, are also showing a flux increase.

Additionally, the model suggests an increased flux through cytidine monophosphate kinase (CMPk) and cytidine diphosphate kinase (CDPk) as well as an increased activity of inorganic diphosphatase (Ppa), converting diphosphate to monophosphate.

The reactions of lower glycolysis are proposed to be upregulated as well. 3-Phospho-D-glycerate (PG3), obtained by the RuBisCO reaction, is converted to 2-phospho-D-glycerate (PG2) by phosphoglycerate mutase (Pgm), to phosphoenolpyruvate (PEP) by enolase (Eno) and finally to pyruvate by pyruvate kinase (PyrK).

To provide the carbon needed for enhanced terpenoid synthesis, the total flux through the Calvin-Benson-Bassham (CBB) cycle is expected to increase. Especially the flux through RuBisCO, fixing CO_2_ as PG3 shows a strong increase. The same applies to the phosphorylation of PG3 to 1,3-bisphosphoglycerate (13DPG) by phosphoglycerate kinase (PgK), its reduction to the MEP pathway precursor G3P by glyceraldehyde 3-phosphate dehydrogenase (Gap2), the conversion to dihydroxyacetone-phosphate (DHAP) by triosephosphate isomerase (Tpi) as well as the subsequent regeneration of D-ribulose 1,5-bisphosphate (RuDP) through the reductive pentose phosphate pathway.

The model predicts an increased flux through the light-dependent reactions of oxygenic photosynthesis in the thylakoid membrane. However, photosynthetic pigments like chlorophyll and carotenoids display a reduced flux. Whereas NADPH production via ferredoxin-NADP^+^ reductase (FNR) is suggested to be increased, ATPase activity is supposed to be decreased. This goes along with a reduced TCA cycle activity.

As enhanced flux through the light reactions and CBB cycle are predicted, photorespiratory metabolism increases as well. However, the model suggests utilizing the glycerate photorespiratory bypass via tartronate semialdehyde. Phosphoglycolate as an inevitable by-product of the photorespiratory chain is converted to first glycolate, then glyoxylate, tartronate semialdehyde and afterwards to glycerate, which can be used to synthesize pyruvate or be recycled into the CBB cycle.

In summary, the model proposes an increased flux through the MEP pathway and terpenoid synthesis as well as the CBB cycle, lower glycolysis and the light-dependent reactions of photosynthesis to enhance squalene production. Additionally, an increased regeneration of the cofactor CTP and a decreased ATP/NADPH ratio are predicted to positively correlate with squalene synthesis. Subsequently, we systematically overexpressed 11 selected targets.

### Systematic overexpression study confirms predictions of FSEOF through altered squalene and pigment content

To test the predictions made by the FBA modeling, 11 genes of interest were inducibly overexpressed in *Synechocystis* Δ*shc* markerless deletion mutant (Dietsch et al., 2021) using the previously characterized rhamnose-inducible promoter system (Behle et al., 2020) including all genes of the MEP-pathway except for *dxr*, which was previously reported to negatively impact terpenoid production upon overexpression (Choi et al., 2016). In addition to genes directly involved in terpenoid synthesis, *gap2*, *pyrK* and *tpi* were overexpressed to increase availability of pyruvate and G3P. All strains, including the control and wild type possessed the pSHDY *rhaS* replicative plasmid. *Synechocystis* Δ*shc* pSHDY *rhaS* serves as the control due to its ability to accumulate squalene. The functionality of the overexpression system was confirmed by performing qRT-PCR on two representative genes, showing increased transcript levels upon induction (**Figure S4** (SI)).

**Figure 2:**
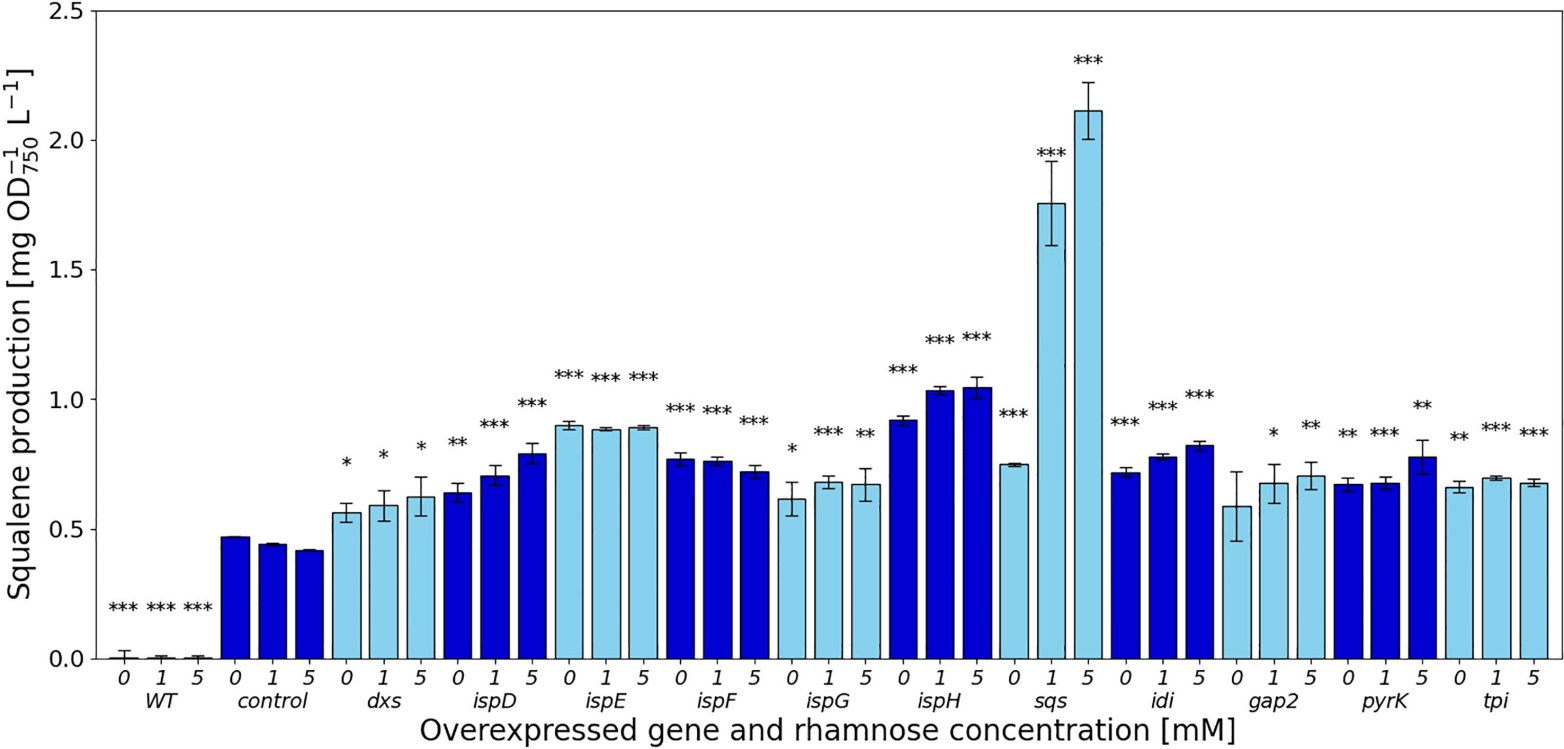
Squalene concentrations [mg l^-1^ OD_750_^-1^] in response to gene overexpressions. Values are represented as the means of three biological replicates, standard deviations are shown. WT represents the *Synechocystis* sp. PCC 6803 wild type, while the control strain is *Synechocystis* sp. PCC 6803 Δ*shc* pSHDY *rhaS*, from which the overexpression strains were constructed by inserting an additional copy of the specified gene under the control of the rhamnose-inducible promoter P_rha_ into its genome. Asterisks (*) represent the p-value of the two-sided t-test between the respective strain and the control strain at the same rhamnose concentration (* denotes a value of p<0.05, ** denotes p<0.01 and *** denotes p<0.001). Samples were measured after three days of incubation with the specified concentration of rhamnose as an inducer.

The experimental work showed that squalene production was increased, albeit in some cases only slightly compared to the Δ*shc* strain for all overexpressed genes identified by FSEOF, as shown in **Figure 2**. The amounts of squalene produced in these strains vary widely, depending on the overexpressed gene (**Table S4** (SI)).

**Figure 3:**
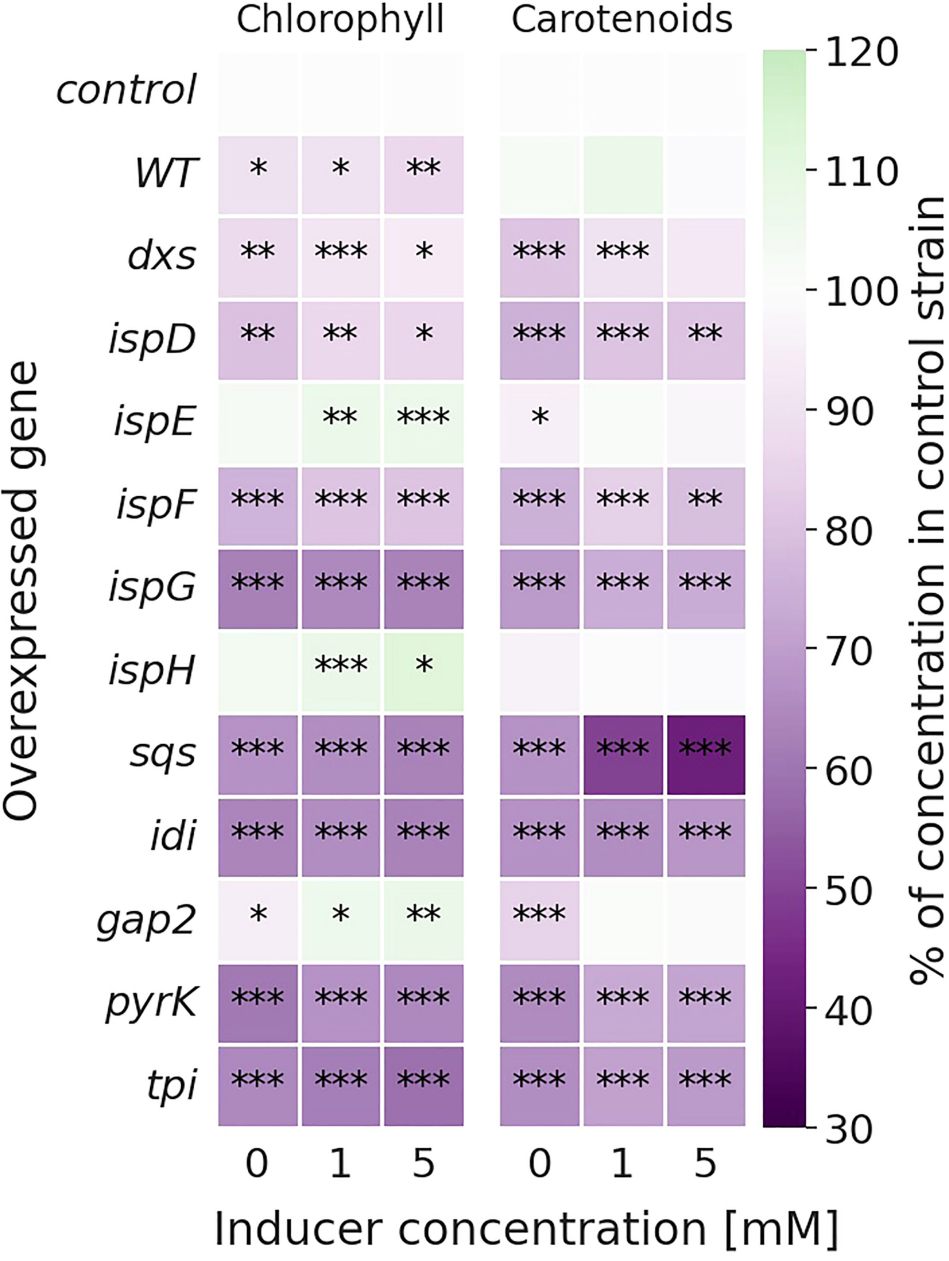
Relative change in chlorophyll (*left*) and carotenoid (*right*) concentrations [mg l^-1^ OD_750_^-1^] of the overexpression strains compared to the Δ*shc* control strain. Values are represented as the means of three biological replicates. WT represents the *Synechocystis* sp. PCC 6803 wild type, while the control strain is *Synechocystis* sp. PCC 6803 Δ*shc* pSHDY *rhaS*, from which the overexpression strains were constructed by inserting an additional copy of the specified gene under the control of the rhamnose-inducible promoter P_rha_ into its genome. Asterisks (*) represent the p-value of the two-sided t-test between the respective strain and the control strain at the same rhamnose concentration (* denotes a value of p<0.05, ** denotes p<0.01 and *** denotes p<0.001). Samples were measured after three days of incubation with the specified concentration of rhamnose as an inducer.

Overexpression of genes strongly altered the pigmentation in several strains, with the relative changes compared to the *Synechocystis* Δ*shc* control strain shown in **Figure 3**. Growth was slightly reduced in the overexpression strains, as shown in **Figure S5** (SI).

**Figure 4:**
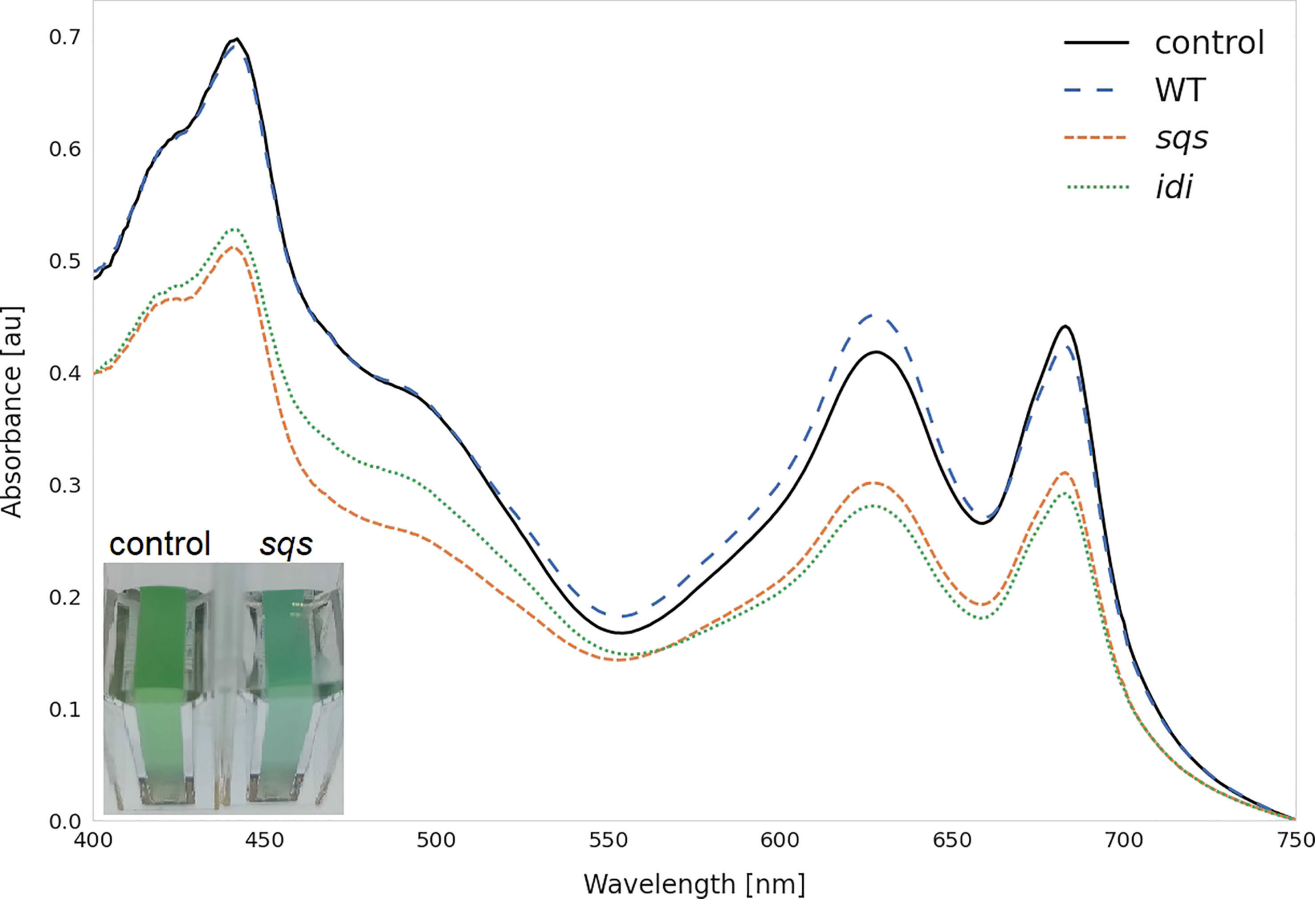
Whole cell spectra of selected strains. Spectra were measured after three days incubation with 5 mM rhamnose in 1 cm cuvettes after equalizing the OD_750_ across all strains and subtracting the OD_750_ as a baseline correction. WT represents the *Synechocystis* sp. PCC 6803 wild type, while the control strain is *Synechocystis* sp. PCC 6803 Δ*shc* pSHDY *rhaS*, from which the overexpression strains were constructed. The visually blue phenotype of the *sqs* overexpression strain in the cuvette is pictured in the bottom left.

Most overexpression strains showed a reduction in chlorophyll and carotenoid content, as shown in **Figure 3** likely due to disruption of optimal pigment synthesis caused by genetic modification, leading to accumulation of metabolites which may inhibit enzymes upstream in their respective pathway. The reduction in pigments may also be caused by the presence of an additional antibiotic resistance cassette in the overexpression strains. However, the strong pigment variations between overexpression mutants suggest only a weak effect of the antibiotic compared to the genetic change. The overexpression of *ispE*, *ispH* and *gap2* are notable exceptions to this observation, showing little changes in pigmentation upon overexpression. Absorbance spectra of all strains can be seen in **Figure S6** (SI). The overexpression of *sqs* led to a blue-colored phenotype, which is characterized by reduced chlorophyll and strongly reduced carotenoid concentrations, as shown in **Figure 4**.

### Overexpression of *sqs* is the most effective way to improve squalene production

In the MEP pathway, overexpression of *dxs* did not have a strong effect on squalene production, shown in **Figure 2**, while the downstream reactions catalyzed by IspD, IspE, IspF and IspH showed more positive effects. The overexpression of both *ispE* and *ispH* led to increased squalene production, even without any inducer present, suggesting that the leakiness of the rhamnose promoter provided sufficient additional enzyme to relieve a bottleneck from the MEP pathway. Overexpressing *ispD* on the other hand increased squalene concentrations in a more concentration-dependent manner. Overexpression of *ispG* did not increase squalene content by a large amount, also showing the strongest negative impact on pigment concentrations of the overexpression of genes in the MEP pathway.

The overexpression of *idi* increased squalene concentrations by 1.96-fold, reducing pigment concentrations in the process, likely by shifting the IPP/DMAPP ratio.

The overexpression of *sqs* led to a 5-fold increased squalene concentration, yielding 2.11 mg OD_750_^-1^ l^-1^. *Synechocystis’* Sqs may be a particularly active enzyme, as cloning in *E. coli* proved challenging, possibly due to toxicity of squalene to *E. coli*, limiting cell densities in liquid culture and favoring mutated or truncated versions of the gene on agar plates. *E. coli* DH5α cells transformed with a non-mutated version of *sqs* typically required around 36 h of incubation at 37°C to form colonies of normal size. The overexpression of *sqs* led to a decrease in chlorophyll and a strong decrease in carotenoid content in *Synechocystis*, as shown by its cell absorbance spectrum in **Figure 4**.

Of the genes not involved in terpenoid synthesis, overexpressing *gap2*, a central enzyme involved in the CBB cycle only led to a small increase in squalene concentrations, but did so while keeping pigment concentrations approximately at the same level as in the Δ*shc* base strain. Upon induction, squalene and pigment content increased with the inducer concentration. Overexpression of *pyrK*, a glycolysis gene, increased squalene concentrations after induction, but reduced pigment concentrations. Expression of *tpi* did not have a strong positive effect on squalene concentrations and also reduced pigmentation.

**Figure 5:**
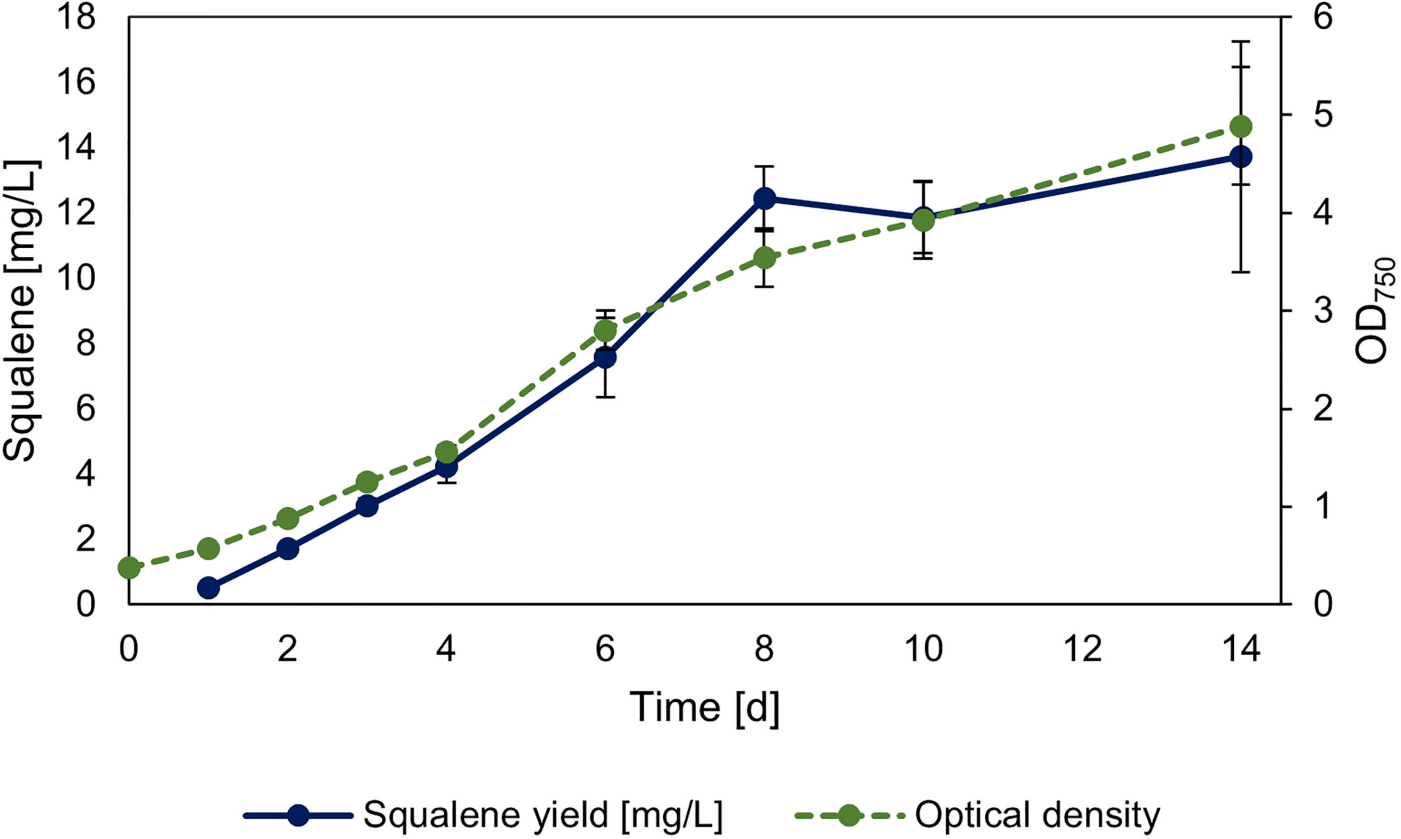
Timeseries of squalene production in *sqs* overexpression strain. Squalene production and OD_750_ of *Synechocystis* Δ*shc* pEERM P_rha_ *sqs* pSHDY *rhaS* in a 30 ml flask culture in mg l^-1^ over a period of two weeks after induction with 5 mM rhamnose to trigger overexpression of the squalene synthase (*sqs*). Means and standard deviations of three biological replicates are shown.

The strain overexpressing *sqs* was additionally investigated regarding its squalene production in 30 ml Erlenmeyer flasks, with its growth and squalene production over time shown in **Figure 5**. The yield of the culture after 14 days was 2.78 mg l^-1^ OD_750_^-1^/13.72 mg l^-1^.

In summary, all overexpression strains showed increased squalene production, with the overexpression of *sqs* leading to a 5-fold improvement compared to the base strain, with longer term cultivation leading to a yield of 13.72 mg l^-1^. The most efficient overexpressions regarding squalene yield were all in the MEP-pathway and terpenoid synthesis, but overexpression of *gap2* was also notable, as it showed a positive impact on both pigment and squalene concentrations with increasing induction.

## Discussion

In the pursuit of identifying potential amplification targets, improving cyanobacterial squalene production upon upregulation, we used a genome scale constraint-based metabolic model. Twelve of the identified overexpression targets were experimentally confirmed.

The analysis suggested an up-regulation of fluxes through the MEP pathway and terpenoid synthesis. Since terpenoids are exclusively synthesized via the MEP pathway in *Synechocystis*, this is very intuitive and shows the robustness of our *in silico* analysis pipeline. Flux through the light-dependent reactions of photosynthesis as well as the CBB cycle were proposed to increase, to meet the demand of fixed carbon and produce the cofactor NADPH. In fact, the FNR reaction showed an increased flux, while the ATPase reaction is proposed to be downregulated, leading to an overall decreased ATP/NADPH ratio. Previous *in silico* studies suggested lower ATP/NADPH ratio requirements for many biofuels as well (Erdrich et al., 2014; Shabestary and Hudson, 2016; Englund et al., 2018). Artificially creating imbalances in this ratio could increase product formation. This can be achieved via different approaches, e.g., by blocking cyclic and other alternative electron flows. As a result, ATP and NADPH are forced to be synthesized exclusively via linear electron flow, whose ATP/NADPH ratio is below the ratio required for biomass synthesis. Knocking out ATP producing reactions or introducing ATP futile cycles or other waste reactions could force the organism to use terpenoid production as a sink for excess reduction equivalents (Erdrich et al., 2014). The carboxylation and oxygenation reaction of RuBisCO were coupled in our model, so the flux through the photorespiratory reactions was forced to increase as well. The model suggested using the glycerate photorespiratory bypass. Nonetheless, since it is an undesired reaction to occur, we did not consider it as an amplification target. On the contrary, Zhou et al. could show that impairing photorespiration leads to a redirection of excess energy and isoprene production could be doubled (Zhou et al., 2021). Additionally, cytidine triphosphate (CTP), a cofactor in the MEP pathway, has to be regenerated by multiple phosphorylations of cytidinmonophosphat (CMP) and the model proposed it might become rate limiting upon high squalene flux. Furthermore, the conversion of diphosphate to monophosphate was predicted to be upregulated because of the increased levels of diphosphate released by IspD, CrtE and Sqs. These results are in accordance with previous *in silico* studies aiming to increase terpenoid production in cyanobacteria, although different models as well as different algorithms were utilized (Lin et al., 2017; Englund et al., 2018). The TCA cycle was indicated to decrease in flux, since it is a sink for carbon, which also was found by previous studies in cyanobacteria (Englund et al., 2018). This finding is in contrast to studies in *E. coli* (Choi et al., 2010), where the TCA cycle is called to be upregulated. This difference can be explained by the fact that heterotrophs like *E. coli* have to regenerate their cofactors via the TCA cycle and autotrophs, in contrast, are able to generate cofactors via the light reactions of photosynthesis. The metabolic model suggested a decrease in photosynthetic pigments as well, since increased squalene production diverts carbon flux away from pigments and they compete for the precursor GPP (Lin et al., 2017).

Flux variability analysis found 31 fluxes to be consistent with the results of the FSEOF analysis and increase upon enforced squalene flux. Among the remaining eight fluxes, three showed a pattern where the maximum flux decreased, while the minimum flux increased, and were thus not considered as amplification targets (Choi et al., 2010). The reaction catalyzed by Idi was the only one to display a sign change, where the maximum flux is positive and the minimum flux is negative, implicating the direction of the metabolic flux is unknown and cannot be determined by flux variability analysis (Flowers et al., 2018). Incorporating kinetic parameters as constraints would help to increase the accuracy of the prediction (Moulin et al., 2021). Since in either direction, the flux through Idi positively correlates with squalene production, we chose to verify the result experimentally. Four reactions were completely unbound, thus providing no further information. Previous studies (Choi et al., 2010) showed that the overexpression of unbound targets also had a positive impact on product synthesis.

To confirm the predictions made by the FSEOF analysis, 11 genes were experimentally overexpressed in *Synechocystis* Δ*shc.* All overexpression strains showed elevated squalene levels, but the degrees to which squalene concentrations were increased varied widely. Overall, growth of the overexpression strains was slightly reduced compared to the control (**Figure S5** (SI)), possibly as a result of the additional antibiotic present in the growth medium. The differences between the overexpression of genes in the MEP pathway are of particular interest, as regulations and feedback mechanisms in the pathway are not entirely known. Since protein tags can affect both activity and stability of enzymes, we chose to use the native protein sequences for overexpression. Since no antibodies are reported for these native enzymes, we were not able to quantify their protein concentrations. However, we confirmed the functionality of our expression system via qRT-PCR for the *dxs* and *sqs* overexpression strains which both showed increased transcript levels upon induction with rhamnose (**Figure S4** (SI)). Quantitative differences in RNA abundance between genes are unavoidable due to differing mRNA lengths and stabilities, but it can be safely assumed that induction of expression through addition of rhamnose yields higher gene expression levels than the control in all cases. Since no protein amounts could be quantified in this study, no conclusions can be drawn regarding the metabolic efficiency of each overexpression relative to the expression strength. Instead, changes in the final metabolite concentrations are caused by the combined effect of the strength of the overexpression and the catalytic activity of the protein.

Dxs is reported to be the rate limiting enzyme of the MEP pathway in many previous studies, but did not prove to be a particularly beneficial overexpression target in this study. This may be attributed to the native Sqs activity not providing a strong enough carbon sink downstream of the MEP pathway, leading to the accumulation of intermediates, such as IPP and DMAPP, which are reported to act as inhibitors to the Dxs enzyme (Banerjee et al., 2013; Álvarez-Vasquez et al., 2021; Di et al., 2022). Kudoh et al., 2017 showed that overexpression of *dxs* led to aggregation of the inactivated protein via allosteric inhibition by IPP and DMAPP, ultimately diminishing the impact of the overexpression on the protein level (Kudoh et al., 2017). Protein inactivation may also explain the small effect of *dxs* overexpression on metabolites in this study.

Other studies reported MEcPP to accumulate upon overexpression of *dxs*, so a dual overexpression with *ispG* may show a more positive effect (Gao et al., 2016; Volke et al., 2019). The overexpression of *ispG* alone did not lead to increased production however, the dependence of IspG on reduced ferredoxin units may be a limiting factor to the conversion of MEcPP to HMBPP (Wang and Oldfield, 2014). If the concentrations of MEcPP were too low for IspG to be limiting, the binding of ferredoxin to the additional enzyme may be the cause of the reduction in photosynthetic pigments, with the overexpression of *ispG* leading to the lowest chlorophyll concentrations among the genes of the MEP pathway.

In contrast to most overexpressions, *ispE* and *ispH* showed a positive effect on squalene production even without inducer present, but not strongly increasing upon induction. This relationship suggests that these enzymatic steps represent a metabolite bottleneck, which can be relieved through a slight overexpression and then yields diminishing returns upon stronger overexpression.

The overexpression of *idi* led to an increase in squalene while decreasing pigments, which is in accordance with the predictions made by the constraint-based model. IspH favors production of IPP from HMBPP over DMAPP, while Idi favors the isomerization of IPP towards DMAPP, leading to an IPP : DMAPP ratio of 3:1 *in vivo*, the optimal ratio for production of GGPP (Chaves et al., 2016; Volke et al., 2019). The overexpression of *idi* likely shifted the IPP : DMAPP ratio in favor of DMAPP.

Overexpressing *sqs* led to the strongest increase in squalene and reduction in carotenoids. Sqs competes with the native GGPP synthase *crtE* for the intermediate FPP, with the overexpression leading to a shift in favor of squalene, away from GGPP, the precursor for carotenoids and phytol. Since there was only little change in growth over three days (**Figure S5** (SI)), the reduced pigmentation did not seem to have a significant negative effect on the cell, but shifting the balance further towards squalene might lead to decreased photosynthetic performance. Overexpression of *ispH* and *ispE* on the other hand increased squalene concentrations as well as the total amount of measured terpenoids, defined as the sum of squalene, carotenoid and the phytol chain of chlorophyll by up to 18%. Combining the overexpressions of *sqs* with *ispH* and *ispE*, may be a promising strategy moving forward, as increased total flux through the MEP pathway can both compensate for the reduced pigmentation caused by *sqs* overexpression and increase squalene titers.

In conclusion, FSEOF allowed us to choose biologically relevant amplification targets computationally, all of which had a positive effect on squalene synthesis upon experimental validation. Considering squalene is synthesized via the linear MEP pathway, most of the identified targets are rather intuitive. Since we were able to confirm all selected targets, we suggest a validation of the non-intuitive targets outside the MEP pathway, such as Ppa and FNR for further studies. These two targets have previously been predicted and tested in *Synechocystis* (Englund et al., 2018). Our findings propose that constraint-based metabolic models could aid in the selection of targets improving the production of desired metabolites. This could be of particular interest for the prediction of combinatorial interventions. However, classic FBA does not account for regulatory mechanisms like feedback inhibition or potential metabolite toxicity (Knoop and Steuer, 2015). It could be helpful for future studies to test FSEOF in combination with dynamic extensions of FBA (Mahadevan et al., 2002) and hybrid kinetic and constraint-based models (Shameer et al., 2022). The incorporation of metabolite concentrations, flux rates or kinetic parameters could drastically improve the precision and reliability of the results (Mahadevan et al., 2002; Moulin et al., 2021; Shameer et al., 2022). For future studies we suggest the combinatorial expression of the identified amplification targets, especially with the native *sqs*, since it seems to be the rate limiting enzyme in the present study, as well as the combination of amplification and knock-down targets. Our experiments are in accordance with the central idea of synthetic biology, where experimental designs are determined by metabolic modeling and experimental results can feed back data into models to increase their accuracy, leading to deterministic, steady improvement. The overexpression of the native *sqs* gene of *Synechocystis* proved to be the most successful strategy for squalene production to date, with the strain reaching a higher production titer than heterologous *sqs* expression in *Synechocystis* (Pattanaik et al., 2020).

## Data availability statement

The original contributions presented in the study are included in the article/**Supplementary Material**. Further inquiries can be directed to the corresponding author.

## Author contributions

AG: Conceptualization, Investigation, Methodology, Computational work, Writing – Original draft, review & editing, Data Visualization. AN: Conceptualization, Investigation, Experimental work, Writing – Original draft, review & editing, Data Visualization. MD: Conceptualization, Methodology, Experimental work, Writing – review & editing. TP: Computational work, Writing -Data visualization, review. DM: Experimental work, Writing – Review & Editing. ST: Experimental work, Writing – Review & Editing. PW: Methodology, Data Curation. IA: Supervision, Writing – review & editing. All authors contributed to the article and approved the submitted version.
