# Supplementary material for: A systematic overexpression approach reveals native targets to increase squalene production in Synechocystis sp. PCC 6803
Source: Front Plant Sci. 2023 May 30;14:1024981. doi: 10.3389/fpls.2023.1024981 (PMC10266222; doi:10.3389/fpls.2023.1024981)
Supplement: Supplementary file 1 [file DataSheet_1.pdf]

Supplementary Material

Supplementary Table 1. Plasmids used in this study and information regarding their origin.

**Plasmid name**	**Source**
pSHDY rhaS	(Behle *et al.* 2020)
pEERM4	(Englund *et al.* 2015)
pEERM4 Prha dxs	This study
pEERM4 Prha ispD	This study
pEERM4 Prha ispE	This study
pEERM4 Prha ispF	This study
pEERM4 Prha ispG	This study
pEERM4 Prha ispH	This study
pEERM4 Prha idi	This study
pEERM4 Prha sqs	This study
pEERM4 Prha crtE	This study
pEERM4 Prha gap2	This study
pEERM4 Prha pyrK	This study
pEERM4 Prha tpi	This study

Supplementary Table 2. DNA sequences of primers used in this study and their modifications for cloning purposes.

Gene	P fwd (5'-3')	P rev (5'-3')	Other modifications
idi	TGACATGGCTAGCGATA GCACCCCCCACCGTAA	AGCCTGCAGTTAAGGTT TAGTTAACCTTT	
dxs	TGACATGGCTAGCCACATC AGCGAACTGACCCACCCCAA TGAG	GCTACTGCAGCTAACTAACTC CAGGAGCGACAACTG	
sqs	TGACATGGCTAGCTCAG GAGTTGATCGCATGAGC	AGCTACTGCAGCTAACTGG CAATAACCCGATTAA	silent mutation in 110L to remove NheI
ispD	TGACATGGCTAGCCATTT ACTAATTCCAGCGGC	GCTACTGCAGTCAGGCGGA TTTTGCCGACC	
ispE	TGACATGGCTAGCCATT CCTACACCCTCCATGCCCCG	GCTACTGCAGTCAATTATTC ATAATTTGGATGCCG	
ispF	TGACGCTAGCACTGCTC TACGCATCGGCAACGG	GCTACTGCAGTTACCCTTCT TTGATTAACAAAGCCACG	
ispG	TGACATGGCTAGCGT AACCGCTTCCCTGCCGACC	GCTACTGCAGTTAAGGGTCA ACCCAACGGC	
ispH	TGACATGGCTAGCGATACCA AAGCTTTTAAACGGTCTCTGC	GCTACTGCAGCTATCCCGCA ATTTCTAGGACG	
gap2	TGACATGGCTAGCACTA GAGTAGCAATTAACGG	GCTACTGCAGCTATTTCCAGTT TTTAGCCAC	silent mutation in 192A to remove NheI
pyrK	TGACATGGCTAGCCAAA CGTCTCCCCTTCCCCGTCG	GCTACTGCAGCTATCCTTTGG ACACCGGGGGTAATGC	
tpi	TGACATGGCTAGCGTGC GAAAAATCATTATTGC	GCTACTGCAGTCAGGGCTGA AAATTAACAA	
***dxs* qPCR**	CCCATACCAGACTAATGGTG ATT	TGCTGAGGCGGACTTTATTT	
***sqs* qPCR**	GCGATCGATGAAGTGGAAGA	CGTCGCACTCTGGAGATTAAG	
***rpoA* qPCR**	CCATGAGTTCGCCACTATTCT	GGCTGATCGGTGTAGCTTT	
**Colony PCR**	ATGCGAATTCGCGGCCGCTTC TAGAG	CTGCAGCGGCCGCTACTAGT ATATAAACGCAGAAAGGCC CACCCGAAGG	Colony PCR primers for insert in pEERM4

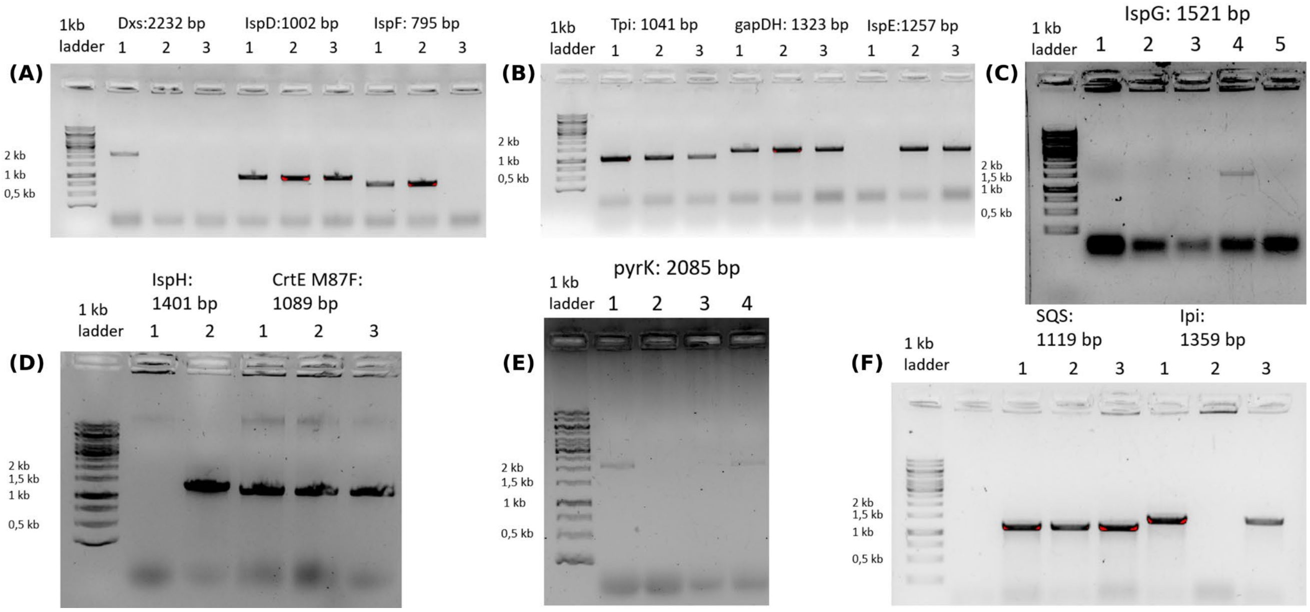

Supplementary Figure 1. Agarose gel electrophoresis of colony PCR products to prove the integration of the respective gene into the genome through heterologous recombination into the neutral site 2 (NS2). Number denote tested colonies, sizes of the expected PCR bands are shown. PCR was carried out with the colony PCR primers shown in Supplementary Table 2 (A) Dxs: 2232 bp, IspD: 1002 bp, IspF: 795 bp (B) Tpi: 2041 bp, gapDH: 1323 bp, IspE: 1257 bp (C) IspG: 1521 bp (D) IspH: 1401 bp, CrtE M87F: 1089 bp (E) PyrK:2085 bp (F) Sqs: 1119 bp, Ipi: 1359 bp

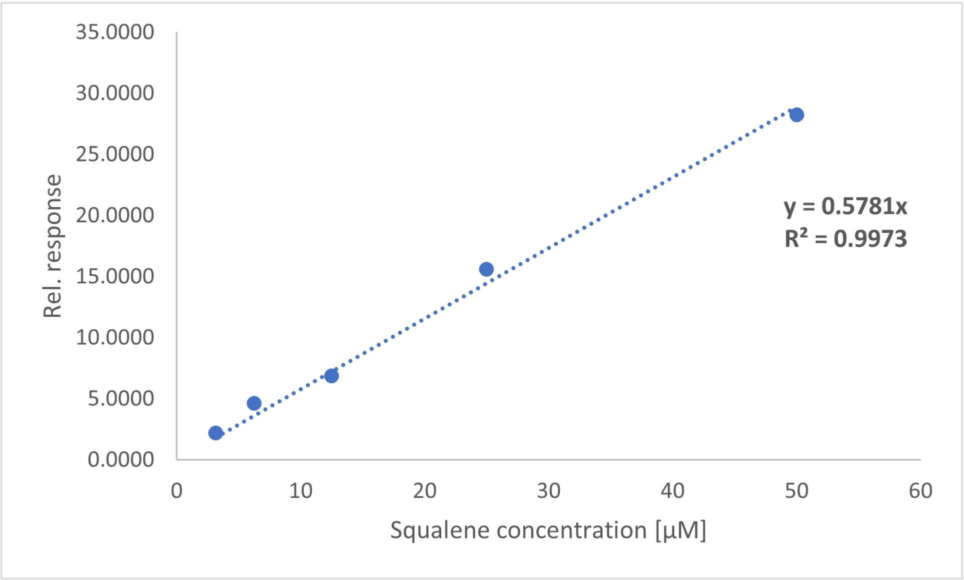

Supplementary Figure 2. GC-MS calibration curve for squalene after extraction of 50, 25, 12.5, 6.25 and 3.125 µM of squalene using the method for squalene extraction from *Synechocystis* cells. Relative response is in relation to the 25 µM β-sitosterol standard, which was solved in the acetone used for extraction.

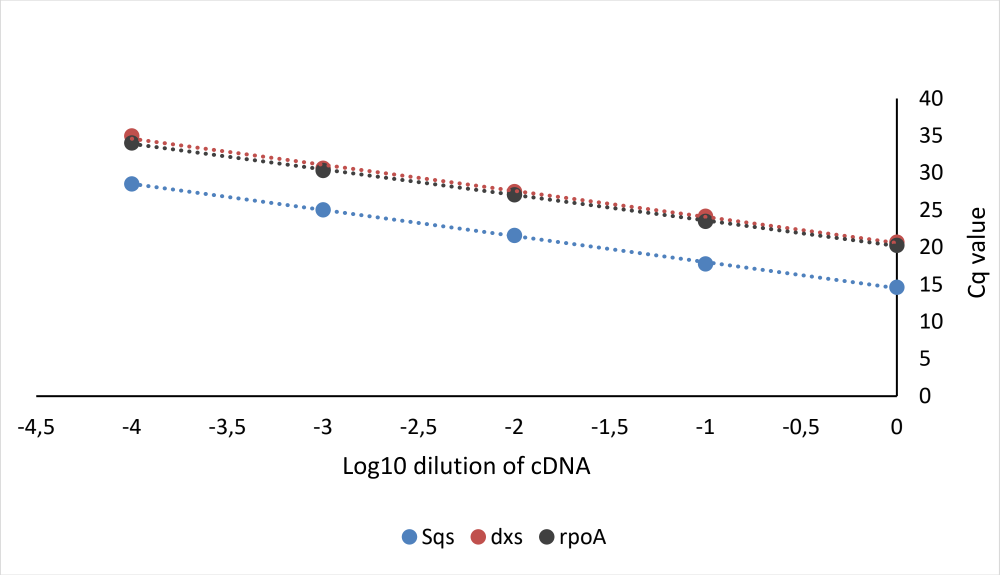

Supplementary Figure 3: Cq values of qRT-PCR primer pairs used with dilution series of cDNA. Primers for *sqs, dxs* and *rpoA* were tested with cDNA extracted after 3 days from *Synechocystis* Δ*shc* pEERM P_{rha} *sqs* pSHDY rhaS, *Synechocystis* Δ*shc* pEERM P_{rha} *dxs* pSHDY rhaS and *Synechocystis* Δ*shc* pSHDY rhaS, induced with 5 mM rhamnose respectively. Primer sequences are shown in Suppl. Table 2.

Supplementary Table 3: Primer efficiencies of qRT-PCR primers used with dilution series of cDNA. Primers for *sqs, dxs* and *rpoA* were tested with cDNA extracted after 3 days from *Synechocystis* Δ*shc* pEERM P_{rha} *sqs* pSHDY rhaS, *Synechocystis* Δ*shc* pEERM P_{rha} *dxs* pSHDY rhaS and *Synechocystis* Δ*shc* pSHDY rhaS, induced with 5 mM rhamnose respectively. Primer sequences are shown in Suppl. Table 2.

**Primer target gene**	**Efficiency**
**Sqs**	**92.96103**
**Dxs**	**92.99725**
**RpoA**	**95.64115**

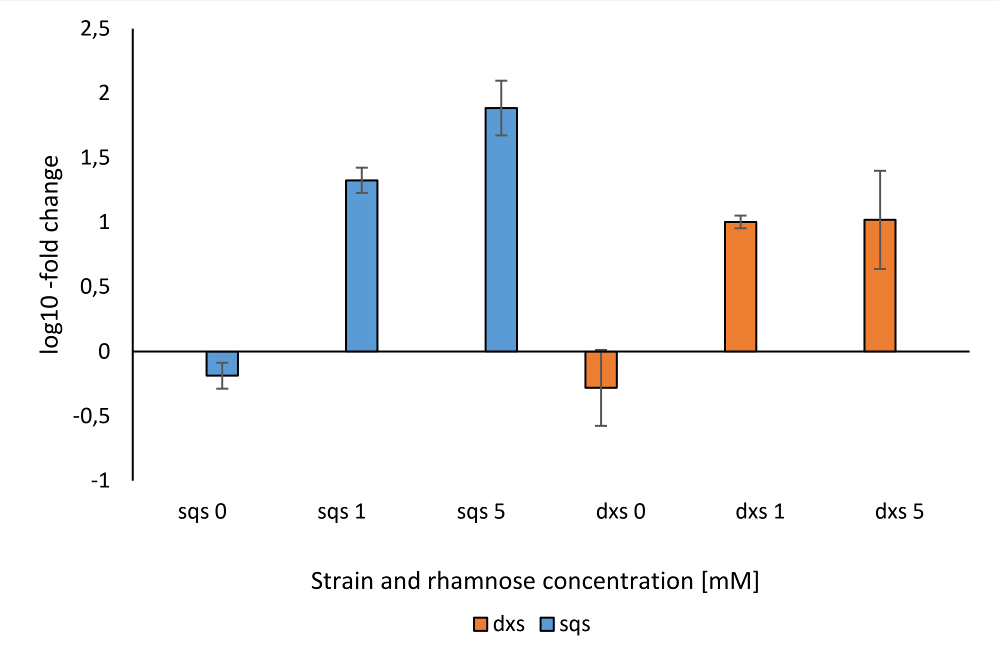

Supplementary Figure 4: Results of qRT-PCR for genes *sqs* and *dxs* as log10-fold changes compared to the control strain in the strains *Synechocystis* Δ*shc* pEERM P_{rha} *sqs* pSHDY rhaS, *Synechocystis* Δ*shc* pEERM P_{rha} *dxs* pSHDY rhaS, respectively. Values were calculated *via* the $2^{-\Delta\Delta C_T}$ method, using *rpoA* as a housekeeping gene and cDNA extracted from *Synechocystis* Δ*shc* pSHDY rhaS, treated with the same rhamnose concentration as a control strain. Primer sequences are shown in Suppl. Table 2. The mean and standard deviation of two biological replicates is shown, which were measured in technical triplicates.

Supplementary Table 4. Squalene yield of all controls and overexpression strains under three different inducer concentrations. WT = Wild type *Synechocystis* sp. PCC 6803, Δshc is the control strain, in which overexpression of the specified genes took place. Mean values and standard deviations of three biological replicates are shown.

**Strain**	**Rhamnose concentration [mM]**	**Yield [mg L-1]**	**Yield [mg OD750 -1 L-1]**	**[mg gCDW-1]**
**WT**	**0**	0.003 ± 0.101	0.001 ± 0.023	0.002 ± 0.058
	**1**	0.01 ± 0.03	0.003 ± 0.008	0.006 ± 0.011
	**5**	0.004 ± 0.06	0.001 ± 0.017	0.003 ± 0.021
**Δ*shc***	**0**	1.5 ± 0.1	0.47 ± 0.03	0.94 ± 0.06
	**1**	1.41 ± 0.02	0.44 ± 0.01	0.89 ± 0.02
	**5**	1.39 ± 0.06	0.42 ± 0.01	0.84 ± 0.02
dxs	**0**	1.47 ± 0.04	0.56 ± 0.04	1.13 ± 0.07
	**1**	1.64 ± 0.22	0.59 ± 0.06	1.18 ± 0.12
	**5**	1.76 ± 0.27	0.62 ± 0.08	1.26 ± 0.15
ispD	**0**	1.85 ± 0.12	0.64 ± 0.04	1.29 ± 0.07
	**1**	1.91 ± 0.15	0.71 ± 0.04	1.42 ± 0.08
	**5**	2.45 ± 0.14	0.79 ± 0.04	1.59 ± 0.08
	**0**	2.53 ± 0.07	0.9 ± 0.02	1.81 ± 0.03

ispE	**1**	2.52 ± 0.03	0.89 ± 0	1.78 ± 0.01
	**5**	2.64 ± 0.03	0.89 ± 0.01	1.79 ± 0.02
ispF	**0**	2.29 ± 0.02	0.77 ± 0.03	1.55 ± 0.05
	**1**	2.28 ± 0.06	0.76 ± 0.01	1.53 ± 0.03
	**5**	2.25 ± 0.08	0.72 ± 0.02	1.45 ± 0.05
ispG	**0**	1.72 ± 0.19	0.62 ± 0.06	1.24 ± 0.13
	**1**	1.98 ± 0.03	0.68 ± 0.03	1.37 ± 0.05
	**5**	2.01 ± 0.2	0.67 ± 0.06	1.35 ± 0.12
ispH	**0**	2.66 ± 0.05	0.92 ± 0.02	1.85 ± 0.03
	**1**	2.86 ± 0.04	1.03 ± 0.02	2.08 ± 0.03
	**5**	2.93 ± 0.17	1.05 ± 0.04	2.1 ± 0.08
sqs	**0**	2.13 ± 0.03	0.75 ± 0	1.5 ± 0.01
	**1**	4.96 ± 0.29	1.76 ± 0.16	3.53 ± 0.33
	**5**	6.23 ± 0.31	2.11 ± 0.11	4.25 ± 0.22
Idi	**0**	2.09 ± 0.09	0.72 ± 0.02	1.44 ± 0.04
	**1**	2.19 ± 0.07	0.78 ± 0.01	1.56 ± 0.02
	**5**	2.52 ± 0.04	0.82 ± 0.02	1.65 ± 0.04
gap2	**0**	1.66 ± 0.36	0.59 ± 0.13	1.18 ± 0.27
	**1**	1.85 ± 0.22	0.67 ± 0.07	1.36 ± 0.15
	**5**	1.98 ± 0.14	0.71 ± 0.05	1.42 ± 0.11
pyrK	**0**	1.94 ± 0.06	0.67 ± 0.03	1.35 ± 0.05
	**1**	1.93 ± 0.05	0.68 ± 0.02	1.36 ± 0.05
	**5**	2.28 ± 0.19	0.78 ± 0.06	1.57 ± 0.13
tpi	**0**	1.84 ± 0.03	0.66 ± 0.02	1.33 ± 0.05
	**1**	2.03 ± 0.06	0.7 ± 0.01	1.4 ± 0.02
	**5**	2.03 ± 0.03	0.68 ± 0.01	1.36 ± 0.03

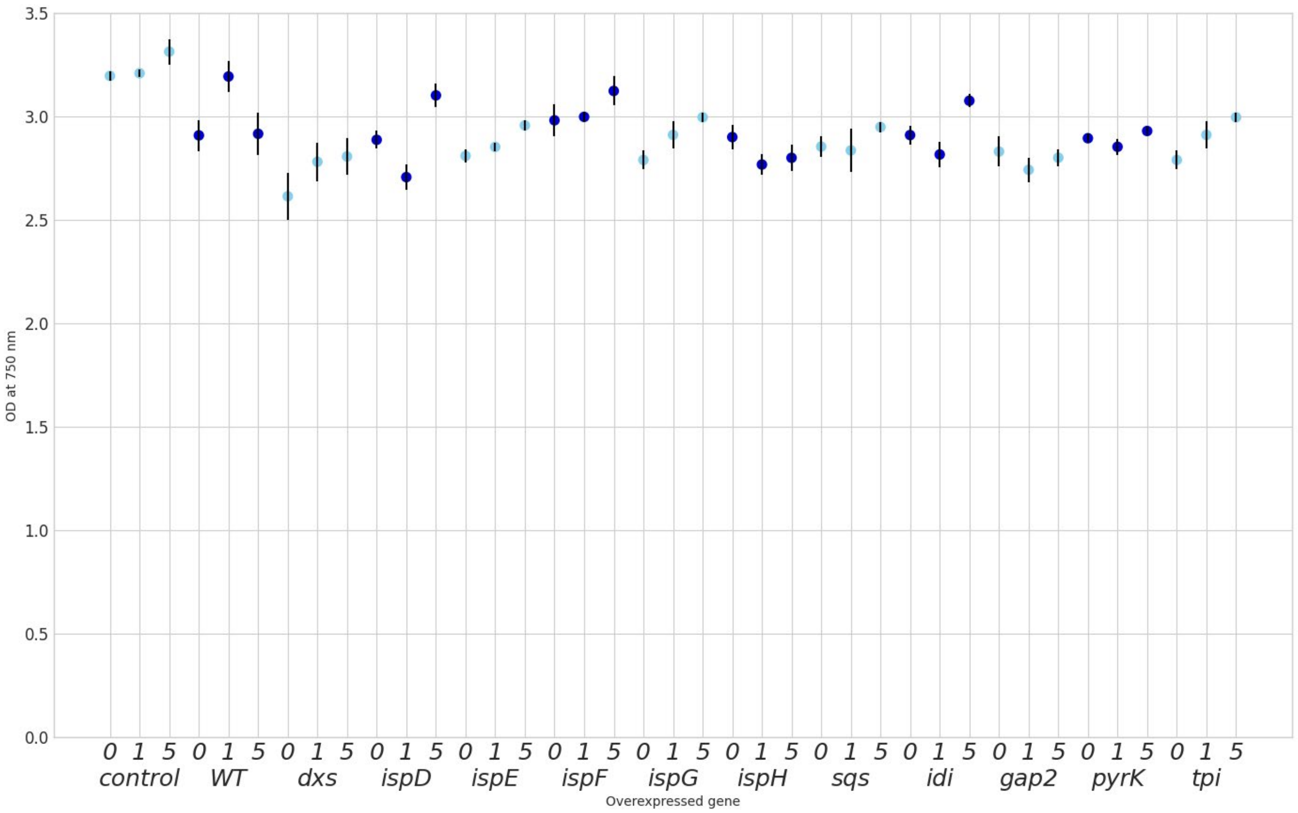

Supplementary Figure 5. Effect of overexpressions on growth of the different strains after 3 days of growth with the indicated rhamnose concentration. Control denotes the Δ*shc* strain in which the overexpression strains were constructed, WT denotes the *Synechocystis* sp. PCC 6803 wild type. Average values from three biological replicates, error bars represent the standard deviation.

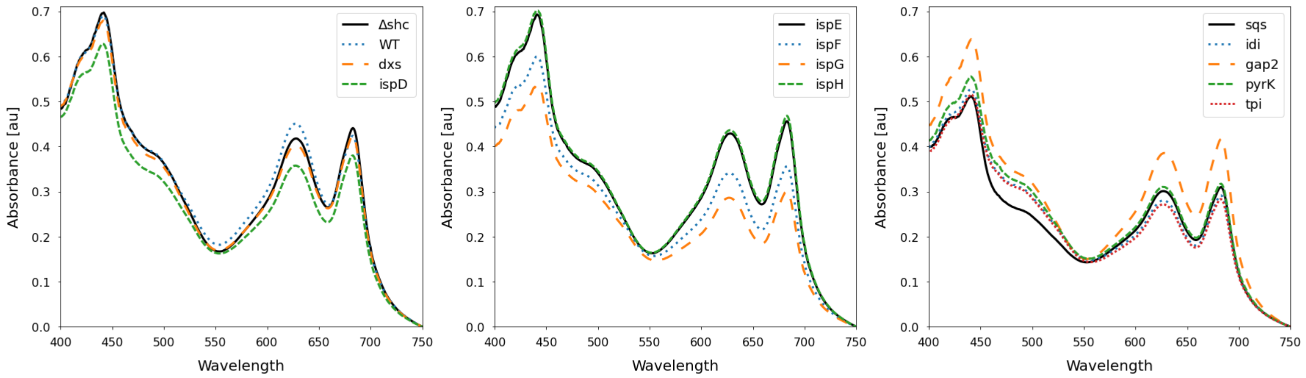

Supplementary Figure 6: Spectra of *Synechocystis* cells after 3 days' incubation with 5 mM rhamnose, measured in 1 cm cuvettes. OD_{750} values were equalized across all measurements in the cuvettes, then the spectra were baseline corrected by subtracting the OD_{750} value.
